# Evaluation of LD decay and various LD-decay estimators in simulated and SNP-array data of tetraploid potato

**DOI:** 10.1007/s00122-016-2798-8

**Published:** 2016-10-03

**Authors:** Peter G. Vos, M. João Paulo, Roeland E. Voorrips, Richard G. F. Visser, Herman J. van Eck, Fred A. van Eeuwijk

**Affiliations:** 1 Plant Breeding, Wageningen University and Research, P.O. Box 386, 6700 AJ Wageningen, The Netherlands; 2 Biometris, Wageningen University and Research, P.O. Box 16, 6700 AA Wageningen, The Netherlands

## Abstract

***Key message*:**

**The number of SNPs required for QTL discovery is justified by the distance at which linkage disequilibrium has decayed. Simulations and real potato SNP data showed how to estimate and interpret LD decay.**

**Abstract:**

The magnitude of linkage disequilibrium (LD) and its decay with genetic distance determine the resolution of association mapping, and are useful for assessing the desired numbers of SNPs on arrays. To study LD and LD decay in tetraploid potato, we simulated autotetraploid genotypes and used it to explore the dependence on: (1) the number of haplotypes in the population (the amount of genetic variation) and (2) the percentage of haplotype specific SNPs (hs-SNPs). Several estimators for short-range LD were explored, such as the average *r* ^2^, median *r* ^2^, and other percentiles of *r* ^2^ (80, 90, and 95 %). For LD decay, we looked at LD_½,90_, the distance at which the short-range LD is halved when using the 90 % percentile of *r* ^2^ at short range, as estimator for LD. Simulations showed that the performance of various estimators for LD decay strongly depended on the number of haplotypes, although the real value of LD decay was not influenced very much by this number. The estimator LD_½,90_ was chosen to evaluate LD decay in 537 tetraploid varieties. LD_½,90_ values were 1.5 Mb for varieties released before 1945 and 0.6 Mb in varieties released after 2005. LD_½,90_ values within three different subpopulations ranged from 0.7 to 0.9 Mb. LD_½,90_ was 2.5 Mb for introgressed regions, indicating large haplotype blocks. In pericentromeric heterochromatin, LD decay was negligible. This study demonstrates that several related factors influencing LD decay could be disentangled, that no universal approach can be suggested, and that the estimation of LD decay has to be performed with great care and knowledge of the sampled material.

**Electronic supplementary material:**

The online version of this article (doi:10.1007/s00122-016-2798-8) contains supplementary material, which is available to authorized users.

## Introduction

Linkage disequilibrium (LD) is the non-random association between alleles at different loci in a breeding population, and can be estimated using the correlation between (SNP) markers when the SNP alleles at those loci are given numerical values, for example, 0 and 1 for bi-allelic SNPs. The amount of LD between loci is important for the success of forward genetic studies, such as Genome-Wide Association Studies (GWAS), because the extent of LD determines the required number of SNP markers and the mapping resolution (Flint-Garcia et al. 2003). Association studies originated from human genetics (Hirschhorn and Daly 2005), where large designed bi-parental populations, such as those used in plants, are impossible. The power of LD-mapping was soon recognized by plant geneticists, and therewith, extensive studies on LD were conducted in *Arabidopsis thaliana* (Nordborg et al. 2002; Kim et al. 2007) and in maize (Yan et al. 2009; Van Inghelandt et al. 2011).

In the classical population genetic sense, the decay with time of LD at generation *t* (*D*_*t*_) is influenced by recombination frequency (*θ*) between two loci and the number of generations of recombination (*t*), since the reference generation *t* = *0* according to the formula *D*_*t*_ = *D*_0_(1 − *θ*)^*t*^. The commonly recognized factors in population genetics, such as non-random mating, selection, mutation, migration or admixture, genetic drift, or a small effective population size, will all affect estimates of LD and LD decay (Flint-Garcia et al. 2003). In heterozygous outbreeders, such as potato, pairs of SNP alleles located on the same haplotype (linked in coupling phase) can display high values of LD and subsequent LD decay is a function of the recombination frequency and the number of generations as described above. In contrast, LD between SNP alleles on different haplotypes (linked in repulsion phase) is not easily detected. For this situation, LD is hard to define and LD falls off to a very low level due to independent segregation of those haplotypes.

Self-fertilizing plants usually show less decay of LD, because in a homozygous genetic background, the recombination events are ineffective to cause LD decay. Accordingly, LD is reported to decay at short distance (100–1500 bp) in an outcrossing crop species, such as maize (Remington et al. 2001; Tenaillon et al. 2001), and at large distance (up to 20 cM) in several selfing crop species, such as barley (Kraakman et al. 2004) or durum wheat (Maccaferri et al. 2005). This is in contrast with natural populations of selfing species, such as *Arabidopsis thaliana* (Nordborg et al. 2002) and *Medicago truncatula* (Branca et al. 2011), where LD estimation suggests a much faster decay of LD (within 10 kb for both species). Perennial or vegetatively propagated species, such as potato and sugarcane, have a long breeding cycle and, therefore, show a limited number of historical recombination events. Hence, LD decays relatively slow (Raboin et al. 2008; D’hoop et al. 2010) in spite of the outcrossing nature of these crops.

Various approaches exist to estimate LD and LD decay. For LD, most approaches are based on the correlation calculated between marker pairs after giving numerical values to allele states, where *r* ^2^ or *D’* is commonly used. LD measures at short range are commonly used in an attempt to robustify LD estimates. Short-range LD is calculated across a certain interval of genetic distances between marker pairs, and then, the mean LD or a percentile of the LD can be used to define the short-range LD. Given a definition for LD, again, various methods can be used to estimate LD decay. Trend lines can be fitted based on LD measures as a function of genetic distance between markers, and this can be done for the mean LD (Yan et al. 2009), the median LD (Myles et al. 2010), or an LD percentile (Adetunji et al. 2014). In addition, the mathematical function for the trend line to describe LD decay can differ. A non-linear regression is most common (Delourme et al. 2013; Stich et al. 2013), but also more flexible functions as the LOESS function (Esteras et al. 2013) and a spline function (Zegeye et al. 2014) have been used. As an alternative to trend lines, thresholds can be defined at which LD stops to exist and we reach equilibrium, i.e., no effective correlation between alleles at different markers. The most commonly used threshold is *r* ^2^ of 0.1, but a threshold of *r* ^2^ = 0.2 has been used as well (Delourme et al. 2013; Li et al. 2014). Adetunji et al. (2014) and Van Inghelandt et al. (2011) used a threshold based on background LD using the correlation between markers from different chromosomes. A further possibility to define an LD decay measure is to identify the distance at which half of the maximum (short range) LD has decayed (Kim et al. 2007; Lam et al. 2010; Branca et al. 2011; Zhao et al. 2011). This value will be referred to as LD_½,90_ in this study and describes the initial slope of the LD-decay curve. Combinations of trend line functions and LD thresholds as described above result in differences in LD-decay estimates, which may severely hinder comparison between studies and species.

The gene pool of potato offers a unique opportunity to unravel the influence of various population genetic factors that affect genome-wide decay of LD. Many varieties have been kept alive by vegetative propagation, and our panel of 537 varieties includes both ancient and modern varieties. In addition, a comprehensive pedigree database (Van Berloo et al. 2007) is available to know the number of generations between varieties of a finite gene pool comprising a few thousand varieties developed over at least two centuries. We used 14,530 SNPs of which we know the physical and genetical positions (Vos et al. 2015), and because every SNP is dated by the year of market release of the variety first showing the SNP variant, we can distinguish the recently introgressed haplotypes from the preceding ones.

In this study, several estimators for LD decay have been explored. To assist our evaluation of these estimators, we also used simulated data, varying in the number of haplotypes (i.e., the amount of genetic variation) and the percentage of haplotype specific SNPs (hs-SNPs). The performance of different LD estimators was compared (average, median, and 80, 90, and 95 % percentiles for the short-range LD), as well as the performance for estimators of LD decay. Special attention was given to estimators for the distance at which half of the short-range LD decayed (*D*_½_). Subsequently, the short-range LD and LD decay were evaluated in a panel of 537 tetraploid varieties, genotyped with a 20K SNP array (Vos et al. 2015). Within this set of genotypes, LD decay was estimated (1) in varieties of different ages to study LD decay over time, (2) within three subpopulations to study the effect of population structure, and (3) using SNPs that have their origin in introgression breeding (admixture). Furthermore, LD decay was estimated using (4) SNPs with different MAF (minor allele frequencies) thresholds and (5) SNPs having different chromosomal positions (in pericentromeric heterochromatin and in euchromatin).

## Materials and methods

### LD-decay estimators and LD-decay estimation

Pearson *r* ^2^ formed the basis for LD estimation. The correlations were calculated on SNP dosage (0–1–2–3–4), both for simulated SNP data as well as for the SNP-array data from the variety panel. The short-range LD was calculated based on markers pairs within 100 kb for the variety panel and within 1 cM for the simulated data. For the short-range LD, five different estimators were used: (1) average of the correlation within the window; (2) 50 % percentile (median); (3) 80 % percentile; (4) 90 % percentile; and (5) 95 % percentile. For both simulated data and real data, all chromosomes were pooled to get a genome-wide LD-decay estimation.

LD decay was estimated using a spline that was fitted on a chosen short-range LD percentile using the RQSMOOTH procedure in GenStat. From the fitted spline, the distance at which half of the short-range LD had decayed was calculated. Typically, the 90 % percentile of the short-range LD was used, LD_½,90_. Background LD was estimated in the varieties panel using 50 randomly chosen markers per chromosome. With these 600 markers, Pearson *r* ^2^ were calculated using all possible marker pairs from different chromosomes. The 95 % percentile of all these pairwise correlations was used to estimate background LD.

### Simulated data

To improve our understanding of LD decay and LD-decay estimators, we simulated a series of tetraploid variety panels using PedigreeSim (Voorrips and Maliepaard 2012). Panels were simulated to resemble the European potato gene pool, as perceived by earlier SNP genotyping studies (Uitdewilligen et al. 2013; Vos et al. 2015). It is assumed that most of the genetic variation present in the contemporary cultivated *Solanum tuberosum* gene pool descends from a limited number of founders, and thus represents a limited set of founder haplotypes (Love 1999). To test the effect of the number of (founder) haplotypes on LD decay, either 6, 8, 10, or 12 founder haplotypes were simulated with allele frequencies ranging from 2.5 to 30 %, according to a geometric distribution Uitdewilligen et al. (2013) (Supplementary file 1). Four haplotypes were randomly assigned to ten tetraploid founder genotypes in generation 0. Initially, the proportion of haplotype specific SNPs (hs-SNPs) was 100 %. With such a simulated data set, we can monitor all recombinations and their effect on LD decay over time. In addition, we varied the percentage of hs-SNPs from the initial 100 % with four additional percentages of 75, 50, 25, and 0 % hs-SNPs, by randomly assigning a SNP to multiple founder haplotypes. A decreasing fraction of hs-SNPs implies an overall increase of the minor allele frequency, because the sequence variant is no longer unique for one of the haplotypes, but is shared by two or more of the 6, 8, 10, or 12 haplotypes. Hence, the fraction of hs-SNPs is confounded with average MAF.

These two variables (4 different numbers of founder haplotypes and 5 different percentages of hs-SNPs) resulted in 20 simulated populations at generation 0, each composed of 10 tetraploid individuals. The simulation of LD decay involved eight generations of random mating, with 200 tetraploid offspring genotypes per generation using the PedigreeSim software (Voorrips and Maliepaard 2012) with eight chromosomes (replicates) of 50 cM each, with 501 SNP markers per chromosome separated by 0.1 cM and a centromere at the 20 cM position, and with random chromosome pairing and a probability of 10 % of quadrivalent formation vs. 90 % of bivalent pair formation. The genotypic scores of the eighth generation were used for calculations on LD and LD decay.

### LD in a panel of 537 potato varieties

In addition to the simulated panels, we evaluated LD and LD decay in a panel of 537 tetraploid varieties and progenitor clones. This panel was genotyped with a 20K SNP array (Vos et al. 2015). These data were analysed using different subsets of marker and/or genotypes in five experiments, as described in the following. In experiments 1 and 2, we make use of subsets of the genotypes and experiments 3, 4, and 5 make use of different subsets of markers.

1. *LD decay over time/generations* It is known that LD decays over generations and distance. To study LD decay in time, the 537 genotypes were assigned to four groups according to age of market release. Group 1 contained genotypes released before 1945 (*n* = 45), group 2 contained genotypes released between 1945 and 1974 (*n* = 42), group 3 contained genotypes released between 1975 and 2004 (*n* = 195), and group 4 contained genotypes released after 2004 (*n* = 255). The age of a genotype is derived from the pedigree database (Van Berloo et al. 2007), where the year of market release was taken. For progenitor clones without market release, we added 10 years to the year the cross was made, as perceived from the first two digits in the seedling code, because in general, it takes ten years between making the cross and naming a variety.
2. *Population structure* Population structure is one of the factors that may influence LD and likewise LD-decay estimates. For this purpose, the population structure was estimated using all markers in STRUCTURE (Pritchard et al. 2000), with an analysis of *K* = 3 groups. Genotypes with a membership probability >0.5 in the STRUCTURE analysis for the “starch” subpopulation (*N* = 59) and the “Agria” subpopulation (*N* = 71) were analysed separately from the large “rest” group (*N* = 407) containing all other genotypes. In addition, population structure was estimated with a principal coordinate analysis with 710 independent markers evenly distributed over the potato genome. Information on the grouping of genotypes is given in Supplementary file 2. Group names are named as described before (D’hoop et al. 2008).
3. *Admixture* In Vos et al. (2015), pre-1945 (old) and post-1945 (new) genetic variants were distinguished. Pre-1945 SNPs represent sequence variants that are polymorphic in varieties released before 1945. The majority of these SNPs continue to be polymorphic in more recent varieties. The new or post-1945 SNPs are monomorphic in old varieties and are, therefore, most likely the result of introgression breeding. Larger LD blocks are expected due to recent admixture with SNPs that originate from donor species. LD decay was analysed separately for pre-1945 and post-1945 SNPs. Old varieties were removed from the latter analysis, because all new SNPs are monomorphic.
4. *MAF* (*minor allele frequency*) *thresholds* LD decay was analysed using SNPs with a MAF of 1.0, 2.5, and 10 %, where MAF >2.5 % is the default set of SNPs, also used in experiments 1 and 2. Variation in MAF thresholds compares well with variation in the number of haplotypes and hs-SNPs in the simulation study to understand the effect of the amount of genetic variation on LD-decay estimates.
5. *Chromosomal position* Recombination is suppressed in pericentromeric heterochromatin and should result in decreased LD decay. Markers located in the pericentromeric heterochromatin, as defined by Sharma et al. (2013), were analysed separately from markers positioned on chromosomal arms (used in experiments 1–4).

The physical distance between markers was extracted from the SNP coordinates on the potato reference genome V4.03 (Sharma et al. 2013). Based on (Sharma et al. 2013), we selected SNPs that were clearly located on the chromosomal arms. For experiments 1 and 2, a subset of 6133 markers was selected of which the minor SNP allele was present at least 5 times in each subgroup (age and subpopulation). The number of markers selected for each experiment on the variety panel is shown in Table 1.

**Table 1:** Number of SNP markers available in various data sets for experiments to evaluate LD decay in a panel of 537 varieties

Experiment^a^	Exp. 1,2 and 4	Exp. 3	Exp. 4	Exp. 4	Exp. 5
Chromosome	MAF ~>2.5 %^b^	New SNPs > 1945^c^ MAF > 1 %	MAF > 10 %	MAF > 1 %	Pericentromeric SNPs, MAF ~>2.5 %
St4.03ch01	780	145	694	969	139
St4.03ch02	773	62	625	957	190
St4.03ch03	489	52	445	644	111
St4.03ch04	575	138	477	787	124
St4.03ch05	548	40	459	793	142
St4.03ch06	470	59	418	586	96
St4.03ch07	550	46	466	639	117
St4.03ch08	456	73	384	585	69
St4.03ch09	424	87	382	568	88
St4.03ch10	316	47	272	401	64
St4.03ch11	435	61	388	592	125
St4.03ch12	317	76	272	405	89
Total	6133	886	5282	7926	1354

^a^Experiments 1–5 are described in the text

^b^Each SNP marker is polymorphic in at least five individuals per age and/or structure group

^c^The SNP markers are only polymorphic in genotypes released after 1945

## Results

### LD decay in simulated data

**Fig. 1:**
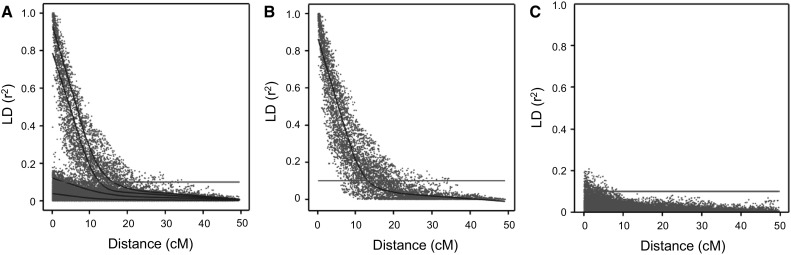
LD decay (in cM) in a simulated data set after eight generations of random mating starting with 100 % hs-SNPs and six founder haplotypes. In **a**, a traditional LD-decay plot is shown. The *blue lines* represent splines fitted for different percentiles (95, 90, 80, and 50 %). from *top* to *bottom*. The LD threshold of 0.1 is indicated with a *red line*. **b** Based on pairwise correlations between marker alleles from the same haplotype. Here, the *blue line* represents the 50 % percentile. In**c**, all pairwise correlations between marker alleles in linked in repulsion phase (different haplotypes) are shown (colour figure online)

As mentioned before, LD in tetraploid potato is mainly the result of physical linkage between two markers. We declare only the pairwise correlations that result from markers in coupling phase of interest for LD and LD-decay estimation. However, in contrast to diploids, where phasing of haplotypes is feasible (Excoffier and Slatkin 1995), phasing information for tetraploid potato is typically lacking. Consequently, LD estimation uses all pairwise allele combinations at marker pairs. This includes correlations between SNP alleles linked in coupling phase, but also less informative correlations between the SNP alleles linked in repulsion phase. To separate the informative (high LD values as a result from linkage in coupling phase) from the non-informative (low LD values as a result from linkage in repulsion phase), we have used simulated data sets with 100 % haplotype specific SNPs with a known phasing of SNP alleles. Using such a data set for conventional LD-decay estimation results in an LD-decay plot, as shown in Fig. 1a. This LD-decay plot contains two kinds of pairwise correlations. Either there is a significant correlation due to the initial linkage between two hs-SNP alleles in coupling phase, or there is immediate linkage equilibrium (LE) due to random chromatid assortment of alleles linked in repulsion phase (i.e., on different haplotypes). However, the known haplotype structure of these data sets allows us to separate the informative pairwise correlation between markers linked in coupling phase from less informative correlations between markers in repulsion phase, as shown in Fig. 1b, c, respectively. The difference between gradual LD decay as a function of genetic distance and immediate linkage equilibrium due to random chromatid assortment is obvious. Figure 1b shows the fitted spline drops below the threshold of *r* ^2^ = 0.1 at a distance of ~13.5 cM. This distance of 13.5 cM appeared to be fairly constant across simulations with 100 % hs-SNPs (Fig. S1). A second important observation is shown in the bottom row of Fig. S2: these graphs show that by adding more haplotypes in a simulated data set also the percentage of non-informative (generally very low) LD values increases and therewith changing the estimation of LD decay using the same estimator. This second observation is in conflict with the standard formula *D*_*t*_ = *D*_0_(1 − *θ*)^*t*^, where the factors *t* and *r* suggest an independence of LD decay with the number of haplotypes in a population. Therefore, we conclude that when we aim at estimating LD and LD decay due to alleles at the same haplotype, the use of all allelic pairs causes a bias that is a function of the number of founder haplotypes, i.e., the genetic diversity.

### Effect of the estimator on LD-decay estimates

In the simulated data, as an estimator for LD decay, we used the intersection of a ‘significance’ threshold (*r* ^2^ = 0.1) and a trend line. The trend lines shown in Fig. 1a and Fig. S2 are based on spline fits on four LD percentiles (50, 80, 90, and 95 %). In addition, LD decay was calculated by the average *r* ^2^, and we looked at D_½,90_ (based on the 90 % percentile). The LD-decay estimates differed greatly among simulated data sets and estimators (Table 2), and rarely reflected the expected value of 13.5 cM for haplotype sharing alleles as described in the previous paragraph. Figure 1a shows that the 50 % percentile (lowest blue line) is always below the threshold of *r* ^2^ = 0.1, and therefore here, in many other simulations, LD decay could not be determined (shown as nd in Table 2). The average *r* ^2^, 50 and 80 % percentiles, always result in a major underestimation of LD decay, while using the higher percentiles (90 and 95 %) resulted in values closest to the earlier determined benchmark value of 13.5 cM. However, the 90 % percentile also failed to estimate LD decay accurately in the data sets with 10 and 12 haplotypes. The simulations demonstrated that the number of haplotypes or the amount of genetic variation had a strong effect on LD-decay estimates. We also show that the use of the LD 90 % percentile resulted in LD decay estimates closest to 13.5 cM when six haplotypes were present and that the 95 % percentile was optimal for 8 or 10 haplotypes. Table 2 suggests that even a higher percentile should be used when 12 haplotypes are present to compensate for the underestimation of LD decay.

**Table 2:** LD-decay estimates (in cM) from simulated data

# Founder haplotypes	% hs-SNPs	Average *r* ^2^	50 % percentile	80 % percentile	90 % percentile	95 % percentile	LD_½,90_
6.0	100	5.7	nd	4.4	12.8	16.0	5.3
	75	5.5	nd	8.8	12.5	14.6	4.7
	50	5.2	nd	9.7	12.6	15.0	3.9
	25	4.8	2.0	9.7	12.3	14.4	4.7
	0	5.1	3.4	10.2	12.7	14.7	4.0
8.0	100	4.2	nd	nd	11.5	14.6	4.9
	75	3.2	nd	4.5	11.0	13.7	4.0
	50	3.2	nd	7.3	11.3	14.1	4.7
	25	2.6	nd	8.1	11.1	13.5	5.3
	0	2.4	0.4	8.8	11.5	13.6	5.0
10.0	100	2.7	nd	nd	3.4	13.9	4.6
	75	2.2	nd	0.8	9.2	13.1	4.1
	50	1.7	nd	5.1	9.9	12.9	5.1
	25	1.1	nd	6.6	10.0	12.2	5.1
	0	1.0	nd	7.6	10.5	12.7	5.5
12.0	100	1.3	nd	nd	nd	12.7	4.3
	75	0.6	nd	nd	7.1	12.0	4.1
	50	0.2	nd	2.8	8.2	11.3	5.4
	25	0.2	nd	5.0	9.1	11.8	5.4
	0	nd	nd	6.1	9.4	11.6	5.4

The values indicate the distances at which the five estimators for LD decay (average *r* ^2^ or a spline of four percentiles (50, 80, 90, and 95 %) drop below the threshold for *r* ^2^ = 0.1. The values shown for D_½_ indicate the distances at which the 90 % percentile decays to half of its initial value. Values in this table represent the average of eight replicates (chromosomes). LD estimates that never exceeded the threshold for *r* ^2^ = 0.1 are not defined (shown as nd). The standard errors of all estimates are very low (average = 0.25)

### Effect of the percentage hs-SNPs on LD-decay estimates

As explained in the previous paragraph, the LD 90 % percentile suits best for the situation with six haplotypes. The five data sets using six founder haplotypes result in fairly similar LD-decay estimates ranging between 12.3 and 12.8 cM. However, the simulated data demonstrate that the different LD-decay estimates are biased in their own way by the percentage of hs-SNPs. The average *r* ^2^ as well as the 95 % percentile provide estimates suggesting a slightly faster LD decay with fewer hs-SNPs. In contrast, the 50 and 80 % percentiles show an opposite bias. Remarkably, the values obtained with the LD_½,90_ estimator do not seem to vary as much as the LD-decay values obtained with any of the other estimators. Therefore, we propose that this estimator is a very promising estimator to compare LD decay across different studies. Unfortunately, the vast majority of LD studies do not yet use LD_½,90_, and the outcome of D_½_ is difficult to compare with other LD-decay estimators.

### Short-range LD in simulated data sets

We observed that LD_½,90_ is the most constant LD-decay estimator in the simulated data sets. The LD_½,90_ estimates rely on an estimation of the short-range LD. The simulations show that the short-range LD estimates (within 1 cM) are also influenced by the number of haplotypes and hs-SNPs. Two remarkable correlations are shown in Table 3. First, the average pairwise SNP correlation is halved with a doubling of the number of haplotypes. Second, the median is decreasing significantly with an increase of the percentage of hs-SNPs. Based on these trends, we can conclude that the amount of genetic variation can be approximated using the average of the short-range LD, and consequently, the optimal percentile can be chosen to estimate LD decay.

**Table 3:** Short-range LD in simulated data sets. Average and median pairwise correlation (*r* ^2^) between pairs of markers within 1 cM

# Founder haplotypes	6 Haplotypes					8 Haplotypes					10 Haplotypes					12 Haplotypes				
% hs-SNPs	100 %	75 %	50 %	25 %	0 %	100 %	75 %	50 %	25 %	0 %	100 %	75 %	50 %	25 %	0 %	100 %	75 %	50 %	25 %	0 %
Average *r* ^2^	0.19	0.19	0.20	0.21	0.22	0.13	0.14	0.14	0.15	0.17	0.10	0.11	0.12	0.13	0.14	0.09	0.09	0.09	0.10	0.11
Median *r* ^2^	0.04	0.07	0.10	0.13	0.15	0.02	0.03	0.06	0.08	0.10	0.01	0.02	0.04	0.06	0.08	0.01	0.02	0.04	0.05	0.06

### Short-range LD in variety panel

Based on the empirical knowledge gained by preceding simulations, it is important to select a suitable estimator for LD analysis in real data. For this purpose, the short-range LD is assessed using pairwise correlations between markers within 1 kb, between 1 and 10 kb, and between 10 and 100 kb. The average *r* ^2^ and 90 % percentile were the highest in the subset of pairwise correlations of markers with 10–100 kb distance, suggesting no LD decay within 100 kb. Therefore, pairwise correlations of markers within 100 kb were used to estimate the short-range LD. These pairwise correlations were pooled over all chromosome arms. Subsequently, the average *r* ^2^ and median *r* ^2^ of the short-range LD were calculated for all experiments except experiment 5 (LD in pericentromeric heterochromatin), as shown in Table 4.

**Table 4:** Short-range LD in variety panel

	Exp. 1. age groups				Exp. 2. structure groups			Exp. 3	Exp. 4. different MAF		
Estimator	<1945	1945–1974	1975–2005	Since 2005	Starch	Agria	Rest	New	MAF > 1 %	MAF (subgroups)	MAF > 10 %
Average *r* ^2^	0.22	0.22	0.20	0.19	0.20	0.21	0.20	0.28	0.14	0.20	0.24
Median *r* ^2^	0.11	0.11	0.09	0.09	0.09	0.10	0.09	0.01	0.03	0.09	0.13
Background LD (*r* ^2^)	0.13	0.14	0.04	0.03	0.10	0.08	0.02	0.07	0.02	0.02	0.02
*D*_½,90_ (Mb)	1.5	1.0	0.8	0.6	0.9	0.8	0.7	2.5	0.8	0.8	0.7

Average and median pairwise correlations (*r* ^2^) between marker pairs within 100 kb in the different experiments on the variety panel. In addition, the 95 % percentile of background LD (between chromosomes) and the distance at which half of the short-range LD is decayed (*D*_½,90_) are shown for each experiment

The average *r* ^2^ ranged between 0.19 and 0.22 for the different age groups and structure groups of varieties (experiments 1 and 2, respectively). For experiment 3, we compared old and new (admixed) variations. The new variation resulted in a higher average *r* ^2^ indicating fewer haplotypes and a low median *r* ^2^ suggesting a high percentage of haplotype specificity. In experiment 4, we compared different MAF thresholds, which resulted in a lower average *r* ^2^ and median *r* ^2^ when more (lower frequent) SNPs were allowed. On average, we find values around 0.2, which is a value similar to what we find in the simulated data sets with 6 haplotypes. In the simulated data sets, the 90 % percentile performed best; therefore, we can conclude that the 90 % percentile will result in a reliable estimate of LD decay in the variety panel. This 90 % percentile was subsequently used to describe LD decay and to calculate the distance, where the short-range LD is decayed by 50 % (LD_½,90_).

### LD decay in different age groups, experiment 1

**Fig. 2:**
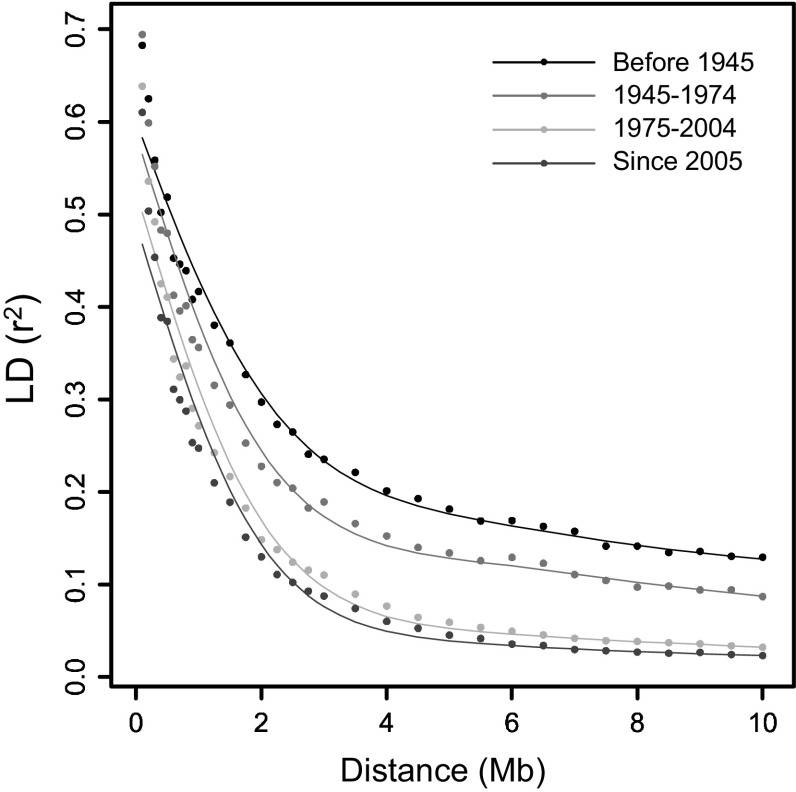
LD decay over generations. The 90 % percentile LD-decay splines are shown for varieties from four different age groups

To evaluate how LD decays over generations within the potato genepool, the variety panel was divided into four age groups. Based on simulated data and short-range LD in the variety panel, a 90 % percentile was used to describe LD decay in the four age groups (Fig. 2). LD decay in individual chromosomes did not show significant differences, and therefore, all chromosomes were pooled. The group with the oldest varieties, released before 1945, displays the most LD (black curve), whereas the group with the youngest varieties, released after 2005, displays the least LD (blue curve). Remarkably, the different age groups decay to a different background level. Therefore, the intersection between the fitted spline and the threshold of *r* ^2^ = 0.1 results in large differences between the age groups, which might not represent the true LD decay. Irrespective of unknown factors influencing background LD, we observe that the slope of all curves flattens between a distance of 2 and 4 Mb. The LD_1/2,90_ values (Table 4) of the different age groups (describing the slope of the first part of the LD-decay curve) may represent the difference within the age groups better than the intersection of the spline with the threshold of *r* ^2^ = 0.1. The group with the older varieties reaches LD_½,90_ at 1.5 Mb and the group of young varieties reaches LD_½,90_ at 0.6 Mb (Table 4), suggesting that in 70 years of breeding, a substantial reduction of LD has been achieved.

### LD decay in different structure groups, experiment 2

**Fig. 3:**
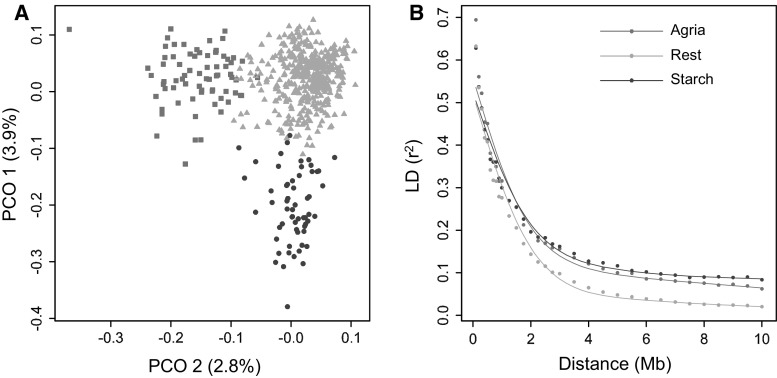
LD decay and population structure. **a** Principal coordinate analysis showing varieties based on >0.5 group membership probability obtained by STRUCTURE.* Green triangles* represent the conventional varieties. *Blue circles* represent modern varieties bred for starch industry. *Red squares* represent modern processing varieties related to Agria. **b** 90 % percentiles show LD decay within subgroups of varieties. *Colour codes* are indicated in the graph (colour figure online)

To estimate the effect of population structure on LD decay, we analysed LD decay within subgroups. Figure 3a shows a principal coordinate (PCO) plot, where the colour of each variety represents a group membership as identified with STRUCTURE (Pritchard et al. 2000). The concordance between the analyses of PCO and STRUCTURE is high, even though the first two dimensions of the PCO explain only 3.9 and 2.8 % of the variation. The red squares identify modern varieties selected for processing industry, and related to the variety Agria. The blue circles identify starch varieties and the green triangles represent all other varieties, mainly fresh consumption. In the principal coordinate and STRUCTURE analyses, additional groups have been considered. The large green group could be separated in a third group with contemporary varieties and a fourth group representing heirloom varieties. The PCO axis separating the contemporary and heirloom varieties explained less than 1 % and was, therefore, not used in this experiment. Figure 3b shows the spline of the 90 % percentile for these three different subpopulations. Again, the curves flatten to different background levels between 2 and 4 Mb. However, the initial slope of these curves shows less difference compared to the age classes resulting in LD_1/2,90_ values ranging between 0.7 Mb for the “rest” group and 0.9 Mb for the “starch” group. Selection for specific market niches resulted in a small reduction of LD decay (Fig. 3b).

### The effect of admixture, MAF threshold, and chromosomal position on LD decay (experiments 3, 4, and 5)

**Fig. 4:**
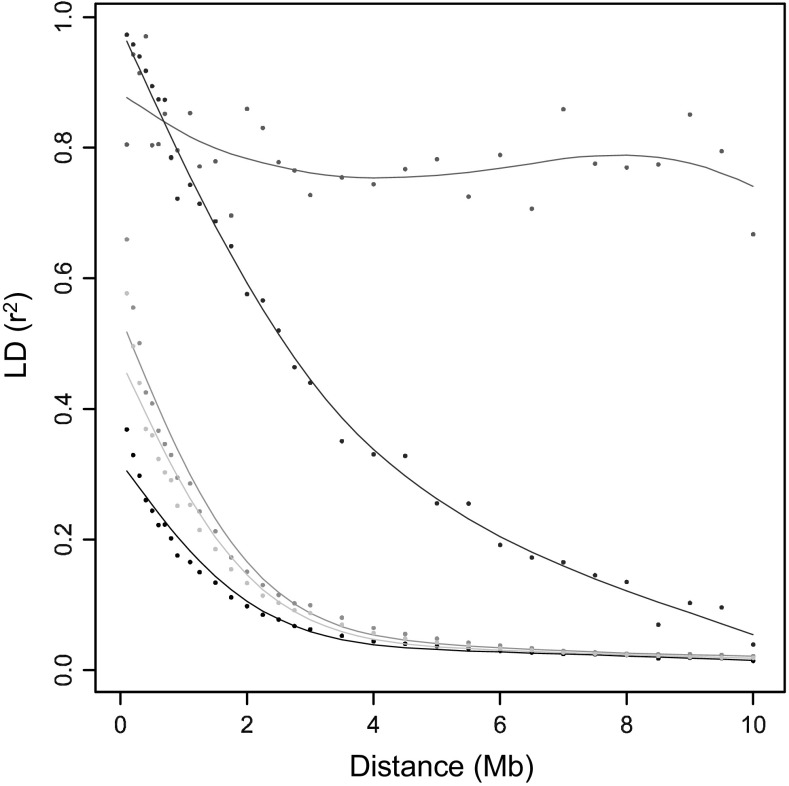
LD decay perceived with different subsets of SNPs. The *blue curve* represents the LD decay between SNPs on haploblocks introgressed since 1945. The *red*, *turquoise,* and *black curves* represent LD decay between subsets of SNPs with different MAF thresholds. *Red* = MAF > 10 %. Turquoise is MAF > 2.5 %. *Black* is MAF > 1 %. The *green curve* represents LD decay between SNPs with physical coordinates located in the pericentromeric heterochromatin (colour figure online)

New sequence variants have entered the potato gene pool due to introgression breeding since 1945 (Vos et al. 2015). The consequences of admixture between wild and elite material on LD decay could be analysed by comparing LD decay among SNPs that are polymorphic in material released before or only after 1945. Figure 4 shows the reduced LD decay perceived between SNPs on introgressed haploblocks (blue curve) that introgressed trait variation (e.g., resistance genes) could be detected with SNPs at several Mb distance, due to large haploblocks.

The black, turquoise, and red curves in Fig. 4 represent LD decay based on the subsets of SNPs with different minor allele frequency thresholds, where the inclusion of more infrequent SNP alleles seemingly results in faster LD decay (black curve) as compared to a more stringent threshold (MAF >10 %, red curve). These curves drop below an *r* ^2^ threshold of 0.1 at significantly different physical distances. In contrast, the D_1/2_ estimates shown in Table 4 are remarkably similar and suggest a decay of LD at distances ranging from 0.7 to 0.8 Mb.

No decay of LD up to 10 Mb was observed between SNPs at physical coordinates that belong to the pericentromeric heterochromatin (Fig. 4 green curve) because of the suppression of recombination in centromeric regions.

## Discussion

Estimation of LD and LD decay is a highly complicated matter without obvious consensus in the literature on the preferred approach. LD decay is influenced by many factors, which usually cannot be analysed separately. In this study, we used simulated and real data representing tetraploid germplasm to understand which factors affect LD and LD-decay estimation. By doing so, we gained more insight in LD patterns in potato and its implications for GWAS and the design of genotyping arrays.

In general, the justification of the number of SNPs on the SNP array is calculated by dividing the total length of a map/genome by the distance, where a genome-wide estimate of LD reaches threshold (often *r* ^2^ = 0.1). In the results, we show that at ~2 Mb, all curves start to flatten. When assuming a genome size of 400 Mb (including the gene rich arms and excluding the pericentromeric heterochromatin (Sharma et al. 2013), we can divide this 400 by 2 Mb, suggesting that 200 markers are required per haploid genome to detect QTLs. However, one could argue that *r* ^2^ of 0.1 is too low for genome-wide QTL discovery. On the other hand, we show that within 100 Kb, no LD decay is observed, and consequently, 4000 markers are needed to cover one haploid genome. In addition to the physical length of haploblocks, also, the number of haplotypes per locus is at stake to determine the number of SNPs on a SNP array. The number (minimum of 200 and maximum of 4000) has to be multiplied by the number of haplotypes in a population. No accurate estimates are known on the number of founder haplotypes in the potato gene pool, but when assuming an average of 10 haplotypes, then 40,000 SNPs is an upper bound for QTL discovery. The 20K SolSTW array, with 14,530 SNPs, does not reach this upper bound, but should still be able to detect sufficient QTLs.

### Simulated data

The main conclusion drawn from simulated data is that estimates for genome-wide values for LD decay depend strongly on the estimator. Up to tenfold underestimations of LD decay were observed (Table 2), which has major implications for the required number of SNP markers for a GWAS, as well as the interpretation of the size of a candidate gene region. A second outcome of this study is the appreciation of the short-range LD values to gain insight in the amount of genetic diversity.

Simulations showed that the average short-range LD values halved when the amount of genetic variation was doubled because of the decreasing signal of linkage disequilibrium from marker pairs in coupling phase. This is caused by the fact that only SNPs residing on the same founder haplotype will result in high pairwise correlations. Correlation between markers on different founder haplotypes will always result in low correlation. Consequently, the percentage of high pairwise correlations will decrease with the introduction of more haplotypes.

### Short-range LD in the variety panel

In the literature, only relatively high averages of the short-range LD values have been reported, for example, in crop species, such as wheat (Würschum et al. 2013) and maize (Yan et al. 2009), where average initial LD of *r* ^2^ = 0.32 and *r* ^2^ = 0.24, respectively, was observed, in (Wang et al. 2013) even averages of *r* ^2^ = 0.5 are observed. We observed a lower average *r* ^2^ for the short-range LD in our data (*r* ^2^ is between 0.19 and 0.22). This suggests that in these studies, either only a limited amount of genetic variation is sampled or these studies dealt with more ascertainment bias compared to what we sampled in potato.

We propose that an estimator for LD decay should be unbiased for, or adapted to the amount of variation present in the gene pool, to allow interpretable comparisons across species. For this purpose, the average of the short-range LD within the variety panel was compared with the short-range LD in simulated data. The observed average *r* ^2^ of approximately 0.2 in the subgroups corresponds well to values observed in simulated data with six founder haplotypes. Therefore, the 90 % percentile is most suitable for analysing LD decay. Indeed, this number of haplotypes is within the range of haplotypes found in the potato germplasm. Earlier haplotyping studies showed five haplotypes of the *LCYe* gene to 16 haplotypes of the *GWD* gene (Wolters et al. 2010; Uitdewilligen 2012).

Different background levels of LD makes it very difficult to determine one threshold at which linkage equilibrium is reached; therefore, we focused on the initial slope of the LD curve and used the LD_1/2,90_ values to compare LD decay within several subsets as described in experiments 1–5.

### LD decay in age classes

To study how LD has decayed over the last century, we compared old and recent varieties. We observed a decrease in LD over the last century from a D_1/2_ of 1.5 Mb in old varieties to 0.6 Mb in the recent varieties, suggesting that haploblocks still have a considerable length. Long haploblocks reflect a breeding history with typically a few meiosis in a century of potato breeding, where newly introduced varieties can be as little as six meioses away from an ancestral variety from the 19th century (Van Berloo et al. 2007). In sexually reproducing crops and natural population, many more meioses take place annually, and therefore, one can imagine that haploblocks in potato stretches much further than in sexually propagated outbreeders. Van Inghelandt et al. (2011) also performed an analysis between old and new genotypes; however, they observe an increase in LD in more recent materials due to fixation for favourable alleles.

### Population structure and LD decay in structure groups

Population structure is a confounding factor, influencing the associations in association mapping and resulting into false-positive associations. Therefore, it is essential to understand the population structure. We observed a weak population structure with PCO1 and PCO2 only explaining 3.9 and 2.8 %, respectively, similar to earlier potato studies (D’hoop et al. 2010; Uitdewilligen et al. 2013). Other studies (Li et al. 2010; Fischer et al. 2013; Stich et al. 2013) report on the absence of significant population structure. The difference between these studies could be explained by the sampling of the Dutch germplasm, where structure groups may result from Dutch breeding efforts. The structure group “starch” is mainly the result from specific breeding of high starch potato varieties within one breeding company. The second group is caused by the frequent use of the variety Agria as parent. Almost every variety within this group has Agria as parent or grandparent and these varieties have all been bred for the processing industry. A higher background LD was observed within these subgroups, as compared to the “rest” group, which could be the result of population structure. LD dropped below the traditional threshold of 0.1 at longer distance within these structure groups, as previously shown by (D’hoop et al. 2010). However, the *D*_1/2_ values showed stable haploblock lengths, ranging from 0.7 Mb to 0.9 Mb.

### Reduced decay of LD due to admixture

Vos et al. (2015) argue that the genetic variants within the potato germplasm can be divided into groups of SNPs predating 1945 and post-1945 variation, based on the year of market introduction of the variety. In this study, LD decay was estimated using pre-1945 and post-1945 SNPs separately. The reduced decay of LD among post-1945 SNPs implies that introgressed haplotypes are substantially longer compared to haplotypes from earlier varieties. Here, we have implicitly defined the length of a haplotype as the physical size of a genomic region flanked by historical recombination events. The dating of SNPs allowed us to quantify the effect of admixture on LD decay. The data suggests that within contemporary varieties, the size of haploblocks is highly variable.

### Effect of MAF on LD decay

In many studies, a restriction on the MAF is applied, where a 5 % cutoff is most commonly used (Zhao et al. 2011; Delourme et al. 2013; Esteras et al. 2013; Wang et al. 2013; Würschum et al. 2013; Adetunji et al. 2014; Li et al. 2014). In some cases, a 10 % (Hyten et al. 2007; Comadran et al. 2011) or even a 20 % cutoff (Branca et al. 2011) is used. In this study, we showed that a restriction on the MAF significantly reduces the average *r* ^2^ and therewith influences the LD-decay estimation when the intersection of a trend line and a threshold is used (Fig. 4). The effect of MAF thresholds was previously shown by (Yan et al. 2009). However, the D_1/2_ values (Table 4) were hardly affected by MAF thresholds.

### Final remarks

Our analyses show that different estimators of LD and LD decay can be chosen, and this choice will result in different estimates of LD decay. In general, we conclude that the LD_1/2,90_ value offers the most consistent estimates of LD decay and performed best in our study. Only a few studies use this estimator (Kim et al. 2007; Lam et al. 2010; Branca et al. 2011; Zhao et al. 2011) and justify a comparative analysis across species. In potato, the distance, where half of the initial LD is decayed, is at least 600 Kb which is substantially longer than values observed in rice (100–300 Kb (Zhao et al. 2011) or 3–4 Kb in *Arabidopsis thaliana* (Kim et al. 2007) and *Medicago trunctula* (Branca et al. 2011). Unfortunately, no earlier study in potato used the LD_1/2,90_ estimator, preventing us to compare our data with the previous estimates of LD decay in potato. On the other hand, a general trend is that background levels of LD are reached at a distance between 2 and 4 Mb. This distance is equivalent to a genetic distance of 5–10 cM, which is in agreement with the 5 cM reported by (D’hoop et al. 2010), and 10 cM reported by Simko et al. (2004). The remarkable low value of LD decay in 275 bp physical distance as reported by Stich et al. (2013) can now be understood as the consequence of the choice for the an LD-decay estimator using the average *r* ^2^ in combination with a non-linear regression.

#### Author contribution statement

Conceived and designed the experiments: PGV, HJvE, and FAvE. Performed the experiments: PGV and REV. Analysed the data: PGV, MJP, and FAvE. Wrote the manuscript: PGV and HJvE. Edited the manuscript: PGV, HJvE, FAvE, and RGFV.

## Electronic supplementary material

Below is the link to the electronic supplementary material.

Fig. S1 LD-decay curves from simulated data with 100% haplotype specific SNPs with 6 haplotypes (left) to 12 haplotypes (right). Only the pairwise correlations are shown resulting from markers that were linked in coupling phase in the founder genotypes (PDF 117 kb)

Fig. S2 LD-decay plots of simulated data underlying the LD-decay estimates shown in Table 2. Each plot represents 1 chromosome of one of the 20 simulated datasets differing in the percentage of haplotype specific SNP and number of haplotypes. In each graphs splines are fitted on four percentile (50%, 80%, 90% & 95%) (PDF 516 kb)

Supplementary material 3 (XLSX 9 kb)

Supplementary material 4 (XLSX 30 kb)
