# Supplementary figures and images for: Evaluation of LD decay and various LD-decay estimators in simulated and SNP-array data of tetraploid potato
Source: Theor Appl Genet. 2016 Oct 3;130(1):123–35. doi: 10.1007/s00122-016-2798-8 (PMC5214954; doi:10.1007/s00122-016-2798-8)

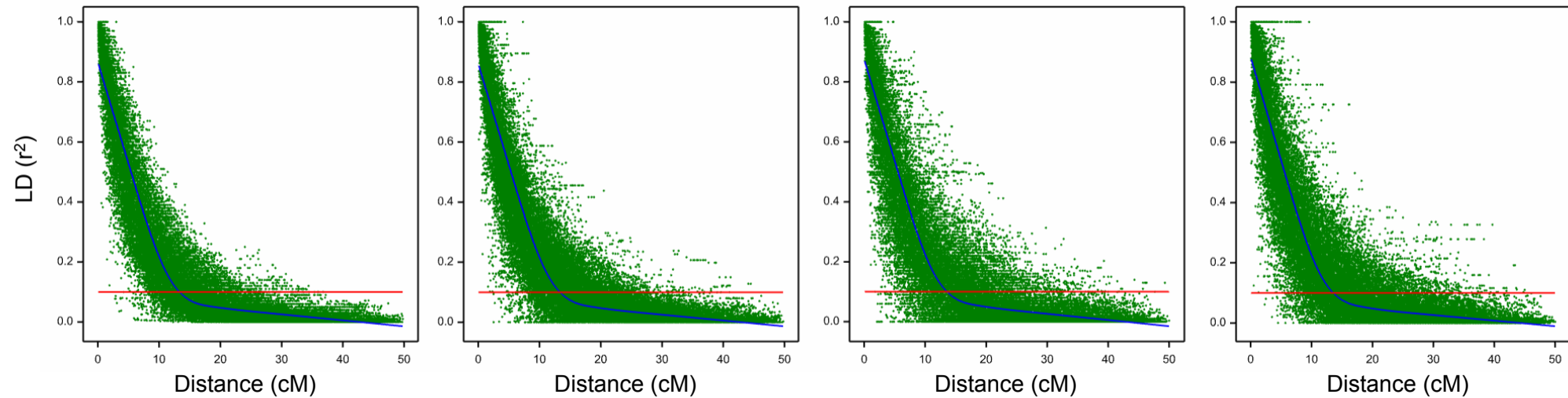

Supplement: Supplementary file 1 — Fig. S1 LD-decay curves from simulated data with 100% haplotype specific SNPs with 6 haplotypes (left) to 12 haplotypes (right). Only the pairwise correlations are shown resulting from markers that were linked in coupling phase in the founder genotypes (PDF 117 kb) [file 122_2016_2798_MOESM1_ESM.pdf]

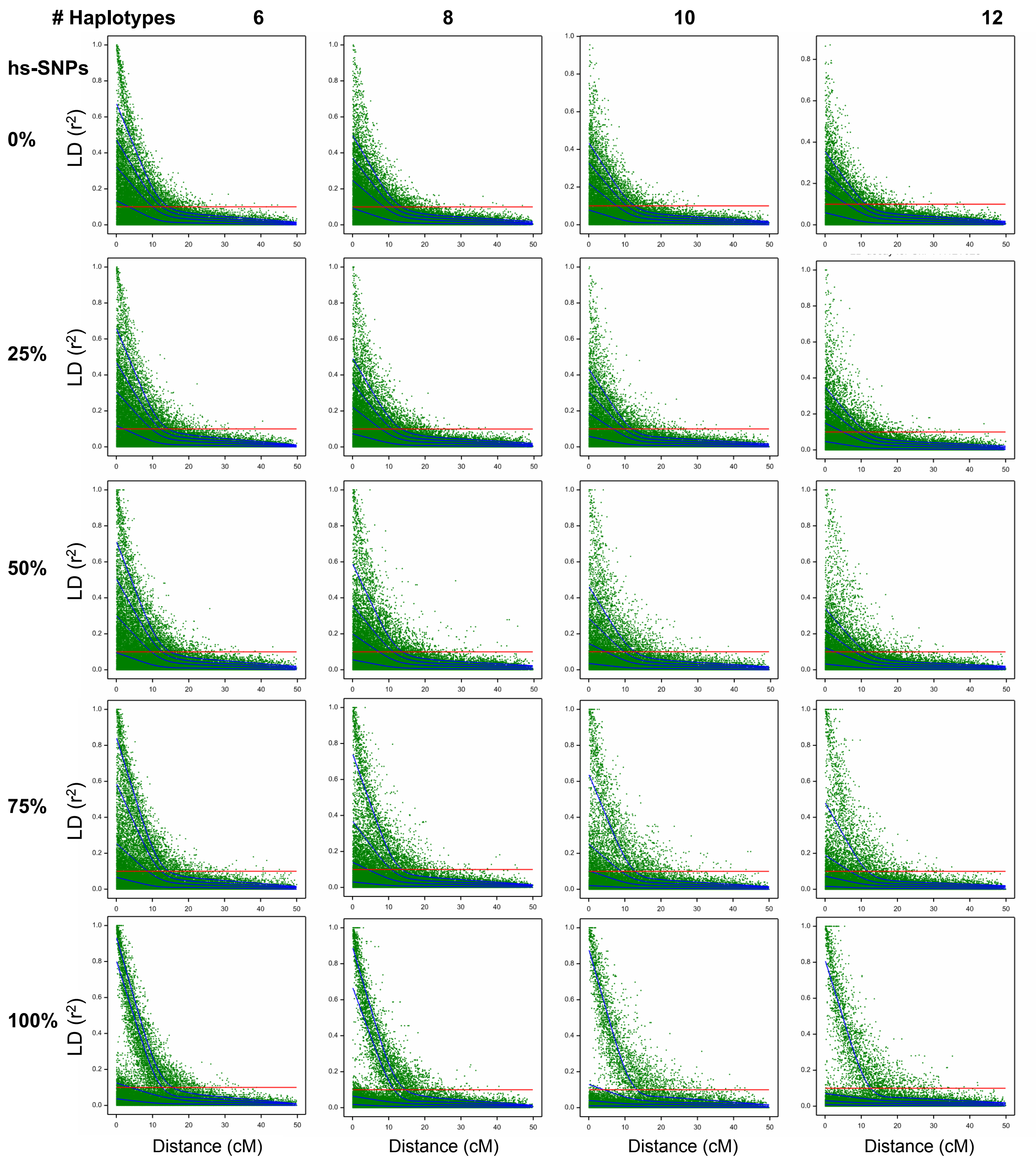

Supplement: Supplementary file 2 — Fig. S2 LD-decay plots of simulated data underlying the LD-decay estimates shown in Table 2. Each plot represents 1 chromosome of one of the 20 simulated datasets differing in the percentage of haplotype specific SNP and number of haplotypes. In each graphs splines are fitted on four percentile (50%, 80%, 90% & 95%) (PDF 516 kb) [file 122_2016_2798_MOESM2_ESM.pdf]
